# Cognitive and Emotional Predictors of Decision-Making in Adults: Roles of Cognitive Functions and Emotion Regulation Difficulties

**DOI:** 10.5964/ejop.17823

**Published:** 2026-08-28

**Authors:** Yuzu Yoshimaru, Shinji Okazaki

**Affiliations:** 1 Graduate School of Comprehensive Human Sciences, University of Tsukuba, Ibaraki, Japan; 2 Institute of Human Sciences, University of Tsukuba, Ibaraki, Japan; 3 Research Fellow of Japan Society for the Promotion of Science, Tokyo, Japan; Stanford University, Stanford, USA

**Keywords:** Iowa Gambling Task, decision-making, cognitive functions, emotion regulation

## Abstract

**Objectives:**

The Iowa Gambling Task (IGT) is a widely used measure of decision-making that mimics real-life situations involving uncertainty and reward. While prior research has separately explored the roles of cognitive functions (e.g., intelligence) and non-cognitive factors (e.g., emotion regulation), few have examined their combined and relative influence. This study aimed to clarify how cognitive abilities and emotion regulation difficulties contribute to decision-making under ambiguity and risk, as assessed by the IGT.

**Methods:**

Fifty healthy adults completed a modified version of the IGT, a battery of cognitive tasks measuring planning, attention, and working memory, and the Japanese version of the Difficulties in Emotion Regulation Scale (J-DERS). Zero-order and partial correlations as well as hierarchical regression analyses were conducted.

**Results:**

Zero-order correlations indicated significant positive associations between advantageous IGT performance and both working memory and attention. These associations remained significant after controlling J-DERS scores in partial correlations. However, hierarchical regression analyses revealed that neither cognitive abilities nor emotion regulation difficulties significantly predicted overall IGT performance. Contrary to expectations, emotion regulation difficulties were not significantly related to risky choices, suggesting a weaker role than previously attributed to emotional intelligence.

**Conclusions:**

The findings suggest that cognitive functions, particularly attentional control, are more strongly associated with advantageous decision-making, especially during later stages of the task. These findings underscore the primacy of cognitive functions in implicit decision-making. Study limitations include a small sample size and a lack of neurophysiological data, highlighting the need for future studies with neuroimaging and larger samples.

The Iowa Gambling Task (IGT; Bechara et al., 1994), one of the most well-known gambling tasks, reflects decision-making processes in everyday contexts (Frank & Seaman, 2023). It is a card-based task in which players repeatedly select one of four cards, “A, B, C, D,” to maximize their monetary gains. Players who choose a card receive a specific amount of money but may also incur a loss. Two of the four card decks provide smaller but consistent gains with lower losses, while the other two offer larger gains accompanied by greater losses. To optimize earnings, players must identify and select the “advantageous” cards, which yield smaller but more stable gains and reduced losses over time. However, players are not informed of the characteristics of each card at the outset, requiring them to gather information and develop an understanding of the task as they progress. Because the consequences of choices are not immediately apparent, the IGT is often referred to as an “implicit gambling task” (Groen et al., 2013). During the initial phase of the task, participants have limited information about the card decks and therefore tend to rely on intuitive or affective responses in their selections. However, as the task progresses, they learn the outcome patterns and begin to rely more on cognitively controlled strategies (Brand et al., 2006, 2007).

Previous research has explored the relationship between IGT performance and both cognitive (e.g., intelligence, cool executive functions) and non-cognitive functions (e.g., emotions, emotion regulation strategies) to identify factors influencing decision-making in situations with rewards (Heilman et al., 2010). Regarding cognitive functions, Colautti et al. (2023) reviewed six studies examining the relationship between cognitive functions and IGT performance in healthy adults. Four of these reported correlations between risky decision-making in the IGT and performance on cognitive tasks. Although some found no significant associations, the overall findings suggest that cognitive functions play a role in decision-making under risk. However, Toplak et al. (2010) indicated that this relationship is less pronounced in studies involving individuals with developmental disorders or other clinical conditions.

The relationship between IGT performance and cognitive abilities is often examined in terms of “hot” and “cool” executive functions. Hot executive functions primarily regulate emotions and reward-related responses, while cool executive functions oversee cognitive control, which aligns with the traditional concept of “executive function” (Zelazo et al., 2005). Although these systems were previously thought to operate independently through distinct neural pathways, recent neuroimaging studies suggest that they interact and co-activate (Le et al., 2019; Nejati et al., 2018; Salehinejad et al., 2021). This highlights the importance of examining how they work concurrently (Moriguchi, 2022).

The relationship between IGT performance and non-cognitive functions, such as emotional intelligence (EI), emotion regulation difficulties, and risk-taking behaviors, has also been explored. For instance, Webb et al. (2014) examined the relationships between EI—measured using the Mayer–Salovey–Caruso Emotional Intelligence Test (Mayer et al., 2003), Bar-On Emotional Quotient Inventory (Bar-On, 2002), and Self-Rated Emotional Intelligence Scale (Brackett et al., 2006)—intelligence (The Wechsler Abbreviated Scale of Intelligence [WASI]; Wechsler, 1997), and IGT performance in healthy adults. Their findings indicated that cognitive functions were more strongly associated with implicit decision-making. Similarly, MacLaren et al. (2023) investigated gambling behaviors via the relationships among risk-taking tendencies, cognitive flexibility (measured by the Wisconsin Card Sorting Task [WCST]), and emotion regulation difficulties (measured by the Difficulties in Emotion Regulation Scale [DERS]; Gratz & Roemer, 2004). Their results showed small but significant negative correlations between risk-taking tendencies and DERS scores, with no significant correlations observed with WCST performance. Additionally, they found that lower sensitivity to negative outcomes was associated with reduced inhibition of risk-taking, particularly among novice gamblers. Further, neurotransmitters have been found to play a substantial role in linking decision-making behaviors in delayed reward contexts, such as the IGT, with both cognitive and non-cognitive functions. For example, dopamine acts on dopaminergic neurons in the prefrontal cortex and is involved in value computations—such as evaluating the risks and costs of rewards—as well as in impulsive decision-making and its inhibition (Soutschek et al., 2023). Additionally, animal studies have shown that increased levels of serotonin promote the selection of larger delayed rewards (Schweighofer et al., 2008).

Together, these findings suggest that both cognitive and non-cognitive functions influence IGT performance. However, few studies have simultaneously examined when and to what extent cognitive and non-cognitive functions contribute to performance on the IGT across its different stages. Moreover, it remains unclear how psychological mechanisms such as difficulties in emotion regulation and attentional processes operate during the transition from ambiguity to risk. Brand et al. (2006, 2007) highlighted that as the task progresses, decision-making requires greater awareness of reward probabilities and loss contingencies. This shift moves decision-making from an ambiguous situation (“decisions under ambiguity”) to a more structured context where risk is better understood (“decisions under risk”). They further suggested that decision-making under risk is more dependent on acquired task information and cognitive functions, whereas intuitive processes, individual differences, and emotions are more influential in decision-making under ambiguity. Colautti et al. (2023) reinforced these findings in their review of studies examining decision-making and executive functions in healthy adults, suggesting the need for future research to account for these shifting situations within the IGT.

Accordingly, the current study aimed to examine the relationship between emotion regulation difficulties and cognitive functions under both ambiguity and risk conditions within the IGT. Our study follows the methodology of Webb et al. (2014), who analyzed the effects of IQ and EI on IGT performance over 20 trials. However, we expand upon their research by focusing on fluid cognitive abilities rather than those acquired through learning and experience. Additionally, rather than concentrating on EI, we investigated measures of emotion regulation difficulties to specifically assess the impact of emotional challenges on decision-making.

We hypothesize that emotion regulation difficulties are more strongly related to risk-taking behavior in “decisions under ambiguity” conditions, with a smaller contribution from cognitive functions. Conversely, in “decisions under risk” conditions, cognitive functions play a more prominent role, while the influence of emotion regulation difficulties is diminished.

## Method

### Participants

Participants included 50 young adults (40 women, mean age [*M_age_*] = 23.24, standard deviation [*SD*] = 4.86). Minimum sample size was determined using G*Power 3.1 (Faul et al., 2009), based on an effect size of 𝑓^2^ = 0.20, power of 1-β = 0.80, and significance level of α = 0.05. All participants were either undergraduate or graduate students from a single university. Individuals diagnosed with any psychiatric or neurodevelopmental disorders, as well as those with sensory function difficulties, were excluded through a screening process. In addition, all participants completed the Conners’ Adult ADHD Rating Scales (Conners et al., 1999) as part of the process. Outliers were identified using boxplots; data points falling outside 1.5 times the interquartile range above the third quartile (Q3) or below the first quartile (Q1) were excluded from the analysis to prevent undue influence on the results. After screening, data from 46 adults (37 women, *M_age_* = 23.35, *SD* = 5.00) were used for analysis.

This study was conducted in accordance with the principles of the Declaration of Helsinki and approved by the Ethics Committee of the Graduate School of Human Sciences, University of Tsukuba (No. 2024-203 A). Before beginning the experiment, the participants received a written and oral explanation regarding the study, after which they gave written informed consent.

### Measures

#### IGT

The IGT (Bechara et al., 1994) is a decision-making task wherein participants aim to maximize their rewards. In line with the original design, the amounts and schedules of gains and losses for each card were pre-set. Participants selected one card per trial across a total of 100 trials. Prior to starting the task, participants were informed that:

- selecting a card would lead to point losses or gains,
- the task would start with 2,000 points, and
- their goal was to select cards to maximize the total points.

The computer version of the IGT was implemented using Python 3.13.2, and PsychoPy ver. 2025.1.0 was used to collect data.

For statistical analysis, the 100 trials were divided into blocks of 10 trials each. We calculated the net score for each block by subtracting the number of disadvantageous card selections from the number of advantageous card selections.

#### Cognitive Factors

To assess cognitive abilities, subtests from the Cognitive Assessment System 2nd edition (CAS2; Naglieri et al., 2014) were administered. Although the CAS2 is a standardized test battery for assessing cognitive functions in children and adolescents, its subtests are based on earlier experimental cognitive tasks and have demonstrated convergent validity in adults (Naglieri et al., 2014). The following subtests were used:

- *Planning.* “Planned Codes” requires participants to strategically fill in matrices with different arrangements based on combinations of letters and symbols. “Planned Connections” resembles the Trail Making Test (Spreen & Gaddes, 1969) planning task and requires participants to connect scattered numbers and letters in the correct order.
- *Attention.* The “Expressive Attention” subtest is similar to a Stroop task (Stroop, 1935) and assesses selective attention under interference, while “Number Detection” involves a visual search for matching numerals, thus requiring sustained attention.
- *Working Memory.* “Verbal-Spatial Relations” requires participants to match the verbal information presented about objects or shapes arranged in a space. For “Sentence Questions,” participants should answer questions immediately following a sequential presentation of information. Each of these tasks is based on the phonological loop and visuospatial sketchpad, two key components of working memory (Baddeley & Hitch, 1994; Georgiou et al., 2008; Naglieri et al., 2014).

The raw score of each subtest were used for statistical analysis.

#### Japanese Version of the Difficulties in Emotion Regulation Scale (J-DERS)

Emotion regulation was assessed using the J-DERS, a self-report questionnaire adapted from the original DERS (Gratz & Roemer, 2004; Yamada & Sugie, 2013). It consists of 16 items that evaluate four factors:

- nonacceptance of emotional responses (Nonacceptance)
- impulse control difficulties (Impulse)
- limited access to emotion regulation strategies (Strategies)
- lack of emotional awareness (Awareness)

Participants rated each item on a 5-point Likert scale ranging from “almost never” to “almost always.” Higher scores indicate greater difficulties in emotion regulation. The sum of scores for each factor was used in statistical analysis.

### Procedure

All experiments were conducted in a university laboratory setting. After providing informed consent, participants completed the J-DERS questionnaire and the computerized IGT. They subsequently completed a paper version of the CAS2 with an examiner. The entire procedure took approximately 90 minutes per participant.

### Statistical Analysis

All statistical analyses were conducted using IBM SPSS Statistics ver. 28. Following the procedure of Webb et al. (2014), zero-order Pearson correlations were calculated between IGT net scores for each block and the CAS2 and J-DERS scores to assess overall relationships. For significant correlations, partial correlations were performed to determine whether the observed effects remained after controlling for either the CAS2 or J-DERS scores.

Hierarchical multiple regression analyses were then conducted to evaluate the predictive power of cognitive and non-cognitive functions on IGT performance. All predictors were entered using the forced entry method. To assess multicollinearity among independent variables, variance inflation factors (VIFs) were calculated. A VIF value exceeding 10 is generally considered indicative of problematic multicollinearity (Hair et al., 2019). In this study, VIF values ranged from 1.14 to 3.83, indicating no significant multicollinearity.

### Data Availability Statement

The behavioral data and analysis syntax used in this study are available on the Open Science Framework (OSF; see Yoshimaru, 2025). Due to copyright restrictions, the questionnaire items from the J-DERS and proprietary materials from the CAS2 are not included in the repository.

## Results

### IGT Performance

**Figure 1:**
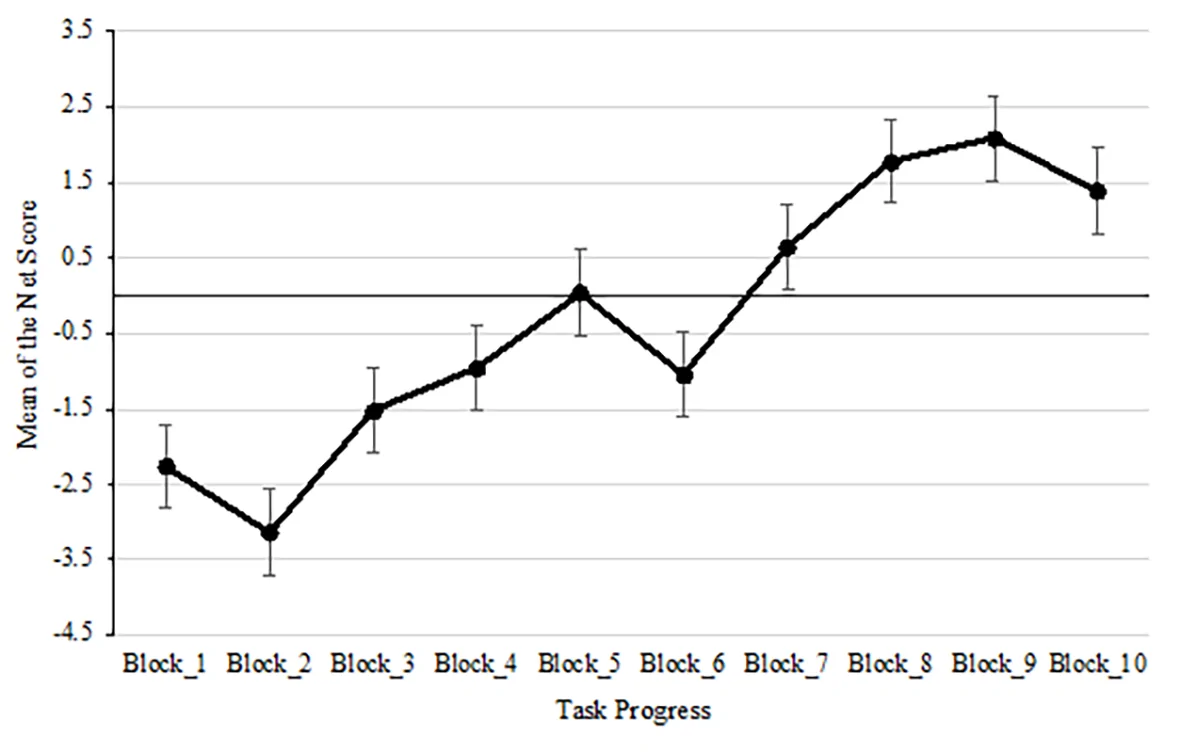
Mean Iowa Gambling Task Performance (±1 Standard Error of the Mean) Across Blocks *Note.* Net scores represent the number of advantageous card selections minus the number of disadvantageous card selections for each block.

Participants’ IGT performance reflected their decision-making under reward. Their IGT net scores were initially low, suggesting poor decisions, but increased in later blocks, indicating a shift toward advantageous choices (see Figure 1). This improvement in the overall net scores suggests that the participants selected more advantageous cards as the task progressed.

### Zero-Order Correlations

Zero-order correlations were calculated to determine the associations between decision-making (IGT) and emotion regulation difficulties (J-DERS) and cognitive abilities (CAS2). The distributions of J-DERS scores and CAS2 raw scores were examined for normality, and no substantial skewness was found. Therefore, no transformations were applied before statistical analysis. The means, *SD*s, and zero-order correlations between variables are shown in Table 1.

**Table 1:** Means, SDs, and Zero-Order Correlations Between Variables

	Mean	*SD*	2	3	4	5	6	7	8	9	10	11	12	13	14	15	16	17	18	19	20
1. Planned code	171.478	22.719	−.410**	.125	.485**	.249	.129	−.055	.039	.007	.278	.066	.043	−.075	.141	.184	−.166	−.045	.023	.089	.135
2. Planned connections	158.261	43.885		−.151	−.490**	−.308*	−.043	.161	.068	.098	−.024	.234	.001	.012	.000	−.023	.084	−.135	−.108	−.133	−.123
3. Expressive attention	99.630	12.524			.002	.117	.030	−.021	.111	.206	.075	−.097	−.037	.066	−.005	.206	.109	−.074	−.001	.123	.032
4. Number detection	143.870	24.292				.177	.266	−.333*	−.257	−.350*	−.152	−.002	.000	−.138	.160	.159	−.012	.351*	.354*	.222	.189
5. Verbal spatial relations	35.022	4.389					.112	.063	−.052	.258	.160	−.188	−.123	−.193	.107	.053	.087	.043	.231	−.037	.228
6. Sentence question	18.304	3.018						−.065	.093	−.085	.008	−.014	.120	.352*	.135	.009	.012	.166	.083	.087	.003
7. J-DERS Nonacceptance	8.565	3.643							.414**	.667**	.489**	−.061	−.074	−.025	−.059	.071	.145	−.026	−.086	.033	.286
8. J-DERS Impulse	9.870	3.619								.661**	.298*	−.187	.059	.096	−.148	−.008	.031	−.085	−.017	.003	−.061
9. J-DERS Strategies	8.739	3.774									.452**	−.217	−.173	.017	−.076	.052	.179	.011	.031	−.011	.190
10. J-DERS Awareness	8.587	2.544										−.102	−.156	.020	−.090	−.074	−.012	−.211	−.166	−.132	.085
11. Block 1	-2.261	3.336											.099	.189	.213	.165	.135	.258	.245	.244	.213
12. Block 2	-3.130	3.667												.237	.315*	.402**	.055	−.020	.035	.350*	−.012
13. Block 3	-1.522	4.004													.192	.232	.297*	.122	.080	.318*	.012
14. Block 4	-0.957	4.894														.505**	.470**	.292*	.096	.290	.161
15. Block 5	0.043	4.371															.496**	.181	.357*	.590**	.308*
16. Block 6	-1.043	5.509																.471**	.516**	.327*	.417**
17. Block 7	0.652	5.367																	.690**	.388**	.466**
18. Block 8	1.783	5.581																		.471**	.398**
19. Block 9	2.087	5.826																			.557**
20. Block 10	1.391	6.020																			

*Note*. *SD* = standard deviation. J-DERS = Japanese version of the Difficulties in Emotion Regulation Scale.

*p < .05. **p < .01.

Significant positive correlations were observed between specific cognitive subtests and IGT performance. Specifically, the CAS2 “Sentence Questions” score (Working Memory subtest) was positively correlated with the IGT net score of Block 3 (*r* = .352, *p* = .016). Additionally, the CAS2 “Number Detection” score (Attention subtest) was positively correlated with the IGT net scores of Blocks 7 and 8 (*r* = .351, *p* = .017; *r* = .354, *p* = .016, respectively). However, no correlation was observed between the J-DERS and IGT scores. To summarize, the results indicate a weak correlation between cognitive functions and decision-making but no correlation between non-cognitive functions and decision-making.

### Partial Correlations

Next, partial correlations were calculated to analyze the associations between IGT performance (net scores on Blocks 1–10) and cognitive task scores. After controlling for all J-DERS factors, the following correlations remained significant:

- “Sentence Questions” (Working Memory subtest) and IGT net score for Block 3 (*r* = .340, *p* = .027)
- “Number Detection” (Attention subtest) and IGT net scores for Blocks 7 and 8 (*r* = .399, *p* = .009; *r* = .390, *p* = .011, respectively).

However, the previously observed correlation between IGT Block 2 net score and “Sentence Questions” (Working Memory subtest) was no longer significant.

Controlling for cognitive functions revealed a significant association between Block 10 performance and scores on the Nonacceptance subscale of the J-DERS (*r* = .363, *p* = .021), suggesting a masked emotional effect during later stages of the task. In other words, the relationship between cognitive functions and decision-making prevailed even after excluding the influence of non-cognitive functions. However, when the influence of cognitive functions was excluded, a relationship that was not initially observed emerged for non-cognitive functions. This suggests that the relationship between non-cognitive functions and decision-making is suppressed by cognitive functions.

### Hierarchical Multiple Regressions

To examine the contributions of cognitive functions and emotion regulation difficulties to IGT performance, hierarchical multiple regressions were conducted. In the first model, we examined whether CAS2 and J-DERS scores explained IGT performance (net scores for Blocks 1–10). In Step 1, CAS2 subtest scores (“Planned Codes,” “Planned Connections,” “Expressive Attention,” “Number Detection,” “Verbal-Spatial Relations,” and “Sentence Questions”) were entered using the forced entry method. In Step 2, J-DERS subscale scores (Nonacceptance, Impulse, Strategies, Awareness) were added to the model.

As shown in Table 2, none of the regression models significantly predicted IGT net scores across any blocks. To confirm these findings, we tested a second regression model. This time, J-DERS scores were entered in Step 1, and CAS2 subtest scores were added in Step 2. Again, as presented in Table 3, none of the models significantly explained the IGT net score. Thus, neither emotional (J-DERS) nor cognitive (CAS2) variables significantly predicted net scores across the 10 IGT blocks. This result indicates that cognitive function as measured by the CAS2 does not exhibit sufficient explanatory power to predict decision-making, even when combined with non-cognitive function as measured by the J-DERS.

**Table 2:** Results of Hierarchical Multiple Regression Analysis Predicting IGT Net Scores, with CAS2 Variables Entered First and J-DERS Variables Added Subsequently

IGT Block	Regression step	Predictor	*R^2^*	Adjusted *R^2^*	Δ*R^2^*	Δ*F*(*df*)	Sig *F*. change (*p* value)
Block 1	1	CAS 2	.117	−.019	.117	*F*(6, 39) = 0.859	.533
	2	J-DERS	.177	−.059	.060	*F*(4, 35) = 0.635	.641
Block 2	1	CAS 2	.042	−.105	.042	*F*(6, 39) = 0.286	.940
	2	J-DERS	.125	−.126	.082	*F*(4, 35) = 0.824	.519
Block 3	1	CAS 2	.252	.137	.252	*F*(6, 39) = 0.186	.065
	2	J-DERS	.267	.058	.016	*F*(4, 35) = 0.187	.943
Block 4	1	CAS 2	.057	−.088	.057	*F*(6, 39) = 0.396	.877
	2	J-DERS	.090	−.170	.032	*F*(4, 35) = 0.311	.869
Block 5	1	CAS 2	.094	−.045	.094	*F*(6, 39) = 0.675	.670
	2	J-DERS	.145	−.099	.051	*F*(4, 35) = 0.521	.721
Block 6	1	CAS 2	.075	−.068	.075	*F*(6, 39) = 0.526	.785
	2	J-DERS	.117	−.135	.042	*F*(4, 35) = 0.417	.796
Block 7	1	CAS 2	.193	.069	.193	*F*(6, 39) = 1.555	.186
	2	J-DERS	.288	.085	.095	*F*(4, 35) = 1.173	.340
Block 8	1	CAS 2	.206	.084	.206	*F*(6, 39) = 1.687	.150
	2	J-DERS	.249	.035	.043	*F*(4, 35) = 0.501	.735
Block 9	1	CAS 2	.076	−.066	.076	*F*(6, 39) = 0.535	.779
	2	J-DERS	.125	−.125	.049	*F*(4, 35) = 0.491	.743
Block 10	1	CAS 2	.080	−.062	.080	*F*(6, 39) = 0.563	.757
	2	J-DERS	.240	.023	.160	*F*(4, 35) = 1.846	.142

*Note.* IGT = The Iowa Gambling Task. CAS2 = Cognitive Assessment System 2nd edition. J-DERS = The Japanese Version of the Difficulties in Emotion Regulation Scale. In Step 1, the CAS2 scores (Planned Codes, Planned Connections, Expressive Attention, Number Detection, Verbal-Spatial Relations, and Sentence Questions) were entered using the forced entry method. In Step 2, the J-DERS scores (Factors 1 to 4) were added to the model.

**Table 3:** Results of Hierarchical Multiple Regression Analysis Predicting IGT Net Scores, With J-DERS Variables Entered First and CAS2 Variables Added Subsequently

IGT Block	Regression step	Predictor	*R^2^*	Adjusted *R^2^*	Δ*R^2^*	Δ*F*(*df*)	Sig *F*. change (*p* value)
Block 1	1	J-DERS	.064	−.027	.064	*F*(4, 41) = .702	.595
	2	CAS 2	.177	−.059	.112	*F*(6, 35) = .796	.579
Block 2	1	J-DERS	.100	.012	.100	*F*(4, 41) = 1.137	.353
	2	CAS 2	.125	−.126	.025	*F*(6, 35) = .165	.984
Block 3	1	J-DERS	.016	−.080	.016	*F*(4, 41) = .164	.955
	2	CAS 2	.267	.058	.252	*F*(6, 35) = 2.003	.092
Block 4	1	J-DERS	.026	−.069	.026	*F*(4, 41) = .279	.890
	2	CAS 2	.090	−.170	.063	*F*(6, 35) = .406	.870
Block 5	1	J-DERS	.024	−.071	.024	*F*(4, 41) = .256	.905
	2	CAS 2	.145	−.099	.121	*F*(6, 35) = .823	.560
Block 6	1	J-DERS	.060	−.031	.060	*F*(4, 41) = .657	.625
	2	CAS 2	.117	−.135	.057	*F*(6, 35) = .374	.891
Block 7	1	J-DERS	.074	−.016	.074	*F*(4, 41) = .819	.520
	2	CAS 2	.288	.085	.214	*F*(6, 35) = 1.758	.137
Block 8	1	J-DERS	.053	−.040	.053	*F*(4, 41) = .570	.686
	2	CAS 2	.249	.035	.196	*F*(6, 35) = 1.526	.199
Block 9	1	J-DERS	.031	−.064	.031	*F*(4, 41) = .324	.860
	2	CAS 2	.125	−.125	.094	*F*(6, 35) = .630	.705
Block 10	1	J-DERS	.142	.059	.142	*F*(4, 41) = 1.700	.169
	2	CAS 2	.240	.023	.098	*F*(6, 35) = .751	.613

*Note.* IGT = The Iowa Gambling Task. CAS2 = Cognitive Assessment System 2nd edition. J-DERS = The Japanese Version of the Difficulties in Emotion Regulation Scale. In Step 1, the J-DERS scores (Factors 1 to 4) were entered using the forced entry method. In Step 2, the CAS2 scores (Planned Codes, Planned Connections, Expressive Attention, Number Detection, Verbal-Spatial Relations, and Sentence Questions) were added to the model.

## Discussion

While previous studies have examined the relationship between IGT performance and both cognitive and non-cognitive functions to identify factors that influence decision-making in reward-related contexts, few have simultaneously investigated the relative contributions of these factors. The present study aimed to fill this gap by examining the relationship between decision-making under ambiguity and risk in the IGT and both cognitive and non-cognitive functions. Following the approach of Webb et al. (2014), we extended prior work by focusing on fluid cognitive abilities and difficulties in emotion regulation. Based on prior findings that decision-making in the early stages of the IGT is more susceptible to emotional reactions and impulsivity (Bechara et al., 1997; Dunn et al., 2006), whereas later stages rely more heavily on cognitive control (Brand et al., 2006, 2007; Fellows & Farah, 2005), we hypothesized that early-stage IGT performance would be more strongly associated with difficulties in emotion regulation, whereas late-stage performance would be more strongly associated with cognitive functions. Our results supported the second hypothesis, indicating that decision-making in the later phases of the IGT was more closely related to cognitive functioning.

### Decision-Making and Cognitive Function

Zero-order correlations showed that the Attention and Working Memory subtests of the CAS2 were weakly associated with advantageous decision-making in the later phases of the IGT. These associations with cognitive functions held even after controlling for J-DERS scores, indicating that cognitive abilities continued to relate to late-stage IGT performance independently of emotion regulation difficulties.

A shift in decision-making from early to later stages of the IGT has been widely documented (Brand et al., 2006, 2007). This change is thought to involve the cognitive process of reversal learning (Rolls, 1999), through which individuals detect evolving patterns of reward and punishment, suppress impulses toward disadvantageous options, and gradually adopt choices that yield long-term benefits. Executing strategies that frequently shift from risky to safer decks—a pattern associated with long-term gain—requires cognitive flexibility and planning (Brand et al., 2007; Noël et al., 2007). Indeed, several studies using the IGT have demonstrated that when task rules and outcome probabilities become clearer in the risk phase, individuals can develop more effective cognitive strategies and more consistently choose advantageous options (Bechara et al., 1997; Crone & van der Molen, 2004). Higher levels of cognitive flexibility and planning have also been linked to more advantageous decisions under risk in the IGT (Ouerchefani et al., 2019).

In this study, we examined the relationship between IGT performance and three cognitive components—planning, working memory, and attention—thereby extending the findings of Webb et al. (2014). Although the association between Working Memory and IGT performance disappeared after controlling for J-DERS, the Attention subtest remained significantly correlated with advantageous decision-making in the later IGT blocks. This pattern suggests that attentional control may play a key role in supporting appropriate decision-making once participants develop a clearer understanding of task contingencies.

Attention is commonly assessed using experimental tasks such as the Stroop task, Go/No-go task, and digit-detection paradigms and is strongly associated with the ability to suppress irrelevant stimuli while selectively focusing on task-relevant information. Although findings on the association between attentional control and performance on reward-based decision-making tasks have been mixed (e.g., Hobson et al., 2011; Xue et al., 2012), attention has been widely recognized as a key component of effective decision-making and adaptive behavior (Miller & Cohen, 2001; Posner & Rothbart, 2007). Prior research has also shown that inhibitory control—which facilitates the suppression of high-risk responses—is involved in decision-making performance (Drechsler et al., 2008; Rastikerdar et al., 2023).

The role of attention and self-regulatory processes in decision-making is deeply intertwined with underlying neurotransmitter systems (Ott & Nieder, 2019; Rotschedl et al., 2024). Among these, dopamine has been identified as particularly important in human delay-reward decision-making (Pine et al., 2010). The behavioral effects of dopamine depend on the specific receptor subtypes it binds to and its interactions with other neurotransmitter systems (Heilbronner, 2017; Soutschek & Tobler, 2023). For example, dopamine binding to D1 receptors (D1R) supports the balance between stability and flexibility within prefrontal networks involved in cognitive functions. In contrast, dopamine binding to D2 receptors (D2R) can sometimes reduce inhibitory control within these same networks. Recent human studies show that blocking D2R leads to reduced delay discounting (i.e., an increased willingness to wait for larger delayed rewards; Soutschek & Tobler, 2023; Wagner et al., 2020). Excessive D2R activation, on the other hand, may destabilize prefrontal networks and has been linked to symptoms such as hallucinations and disorganized thinking observed in people with schizophrenia (Mueser & McGurk, 2004).

Cognitive functions relevant to decision-making—such as self-control and attention—are fundamentally supported by the activity of neurotransmitter systems, especially dopamine. When dopaminergic activity remains in a heightened state, prefrontal regulatory processes may weaken, increasing the likelihood of selecting high-risk options. While the present study identified associations between cognitive functions and decision-making behavior, neurotransmitters serve as the foundational biological mechanisms shaping human decision patterns and play an active role in guiding behavioral regulation.

Furthermore, the present study demonstrated that these associations between cognitive functions and decision-making remained significant even after accounting for non-cognitive factors. However, the role of cognitive functions observed in this study appeared to be more limited than that reported by Webb et al. (2014), as reflected in the smaller Δ*R*^2^ values. Rather than being driven by specific cognitive components, decision-making in the IGT may be influenced more holistically by intellectual ability acquired through learning and experience, such as that measured by the WASI.

Although multiple studies have reported associations between IGT performance and cognitive functions, few have examined their relative contribution in comparison with non-cognitive functions. The present study demonstrated that even after accounting for non-cognitive factors, appropriate decision-making in the later stages of the IGT—when participants have developed a clearer understanding of task contingencies—was particularly associated with attentional inhibitory control. This finding supports prior work, including that of Webb et al. (2014), that emphasizes the importance of cognitive functions in decision-making. Previous research has reported inconsistent results regarding the relationship between attentional abilities and decision-making, with some studies questioning the extent to which cognitive functions contribute to reward-based decision-making (Toplak et al., 2010). However, by showing that attentional control remains a significant predictor even when non-cognitive factors are controlled for, the present study provides important evidence for the contribution of cognitive functions to decision-making processes.

### Decision-Making and Non-Cognitive Function

Zero-order correlations indicated that J-DERS was not associated with IGT performance. The correlations between J-DERS scores and IGT performance in both the early and later stages were not as strong as those reported by Webb et al. (2014). These findings suggest that difficulties in emotion regulation may play a smaller role in IGT decision-making compared to EI. Thus, individuals with emotion regulation difficulties do not necessarily make inappropriate decisions in the IGT. The weaker relationship between J-DERS and IGT performance, compared to EI, may be due to the broader spectrum of emotional management abilities measured by EI, including emotional maturity and interpersonal skills (e.g., Mikolajczak et al., 2006). These abilities will likely contribute to regulating emotional and rational choices during the IGT. Because EI captures a multifaceted ability to regulate emotions, its association with IGT decision-making may be more pronounced.

Moreover, in the present study, controlling for cognitive functions revealed significant associations between non-cognitive factors and decision-making that had not emerged in zero-order correlations. This pattern suggests that cognitive functions acted as a suppressor variable, masking the relationship between non-cognitive factors and IGT performance. This finding contrasts with the pattern reported in Webb et al. (2014), where significant zero-order correlations diminished substantially once cognitive functions were controlled. Such a pattern implies that cognitive functions operated as a confounding or mediating variable in their study. Despite these contrasting statistical patterns, both sets of findings converge on one conclusion: cognitive functions influence the relationship between non-cognitive factors and decision-making. Although only a limited number of studies have simultaneously examined cognitive and non-cognitive functions and decision-making in reward-based contexts, the results from the present study and those reported by Webb et al. (2014) highlight the importance of cognitive abilities in shaping decision-making processes under conditions involving rewards.

### Hierarchical Multiple Regression Analysis

The regression models using J-DERS and CAS2 did not significantly predict IGT performance, yielding results that diverge from those reported by Webb et al. (2014). One possible explanation is that the present study conceptualized cognitive and non-cognitive functions in a narrower sense—focusing on fluid intelligence and emotion regulation—compared to the crystallized intelligence and EI examined by Webb et al. (2014). Given that decision-making is influenced by a wide range of individual factors, including experience and learning capacity (Simon, 1955; Tversky & Kahneman, 1974), broader measures of intelligence may provide greater explanatory power.

Moreover, reward-based decision-making is supported by interactions among multiple brain regions, including the medial and lateral prefrontal cortex, orbitofrontal cortex, anterior cingulate cortex, and several subcortical limbic structures such as the amygdala–hippocampal complex and the sensorimotor territories of the dorsal striatum (e.g., putamen, caudate; Salehinejad et al., 2021). In addition, interoceptive signals—such as cardiac or electrodermal responses mediated through the autonomic nervous system—have also been shown to influence decision-making (Dunn et al., 2010).

Because of these complexities, decision-making cannot be fully explained by behavioral indices of cognitive or non-cognitive functions alone. Instead, the functional brain networks underlying these capacities, as well as environmental and physiological factors, also contribute to decision-making under reward. While the present study assessed cognitive and non-cognitive functions behaviorally, future research should incorporate measures of neural activity and physiological responses to more comprehensively identify the mechanisms that support reward-based decision-making.

### Limitations

This study has several limitations. The first is its small sample size. Based on the G*Power calculation, we aimed to achieve adequate statistical power to detect medium to large effect sizes. However, considering the current concerns regarding reproducibility in psychological research and the need to establish more robust evidence, the sample size of the present study cannot be considered sufficient. With a sample size of 46, there is a possibility that the study may lack sufficient power to detect small effect sizes, which could affect the generalizability and robustness of the findings. A small sample size increases the absolute value of the test statistic required to reject the null hypothesis, which can lead to situations in which the null hypothesis is retained even when it is false. This reflects a reduction in statistical power and consequently increases the risk of Type II errors (β). Therefore, the results of this study should be interpreted with these limitations in mind, particularly regarding the precision of effect size estimation and the replicability of the observed effects. Future studies with larger sample sizes would help to validate and expand on the current findings, enhancing the external validity and statistical power of the results. As Brysbaert (2019) notes, studies with limited sample sizes can enhance statistical power by increasing the number of observations per participant, thereby producing more stable estimates. It is also necessary to examine specific within-subject effects—such as main effects in within-participant conditions—to strengthen the reliability of the conclusions.

Second, other factors related to decision-making must also be considered. Multiple regression analysis results showed that cognitive functions alone could not explain the differences in decision-making. Decision-making involves various modalities, such as brain function and biological reactions, that should be included in future studies.

Lastly, the relationship between IGT performance and both cognitive and non-cognitive functions was examined using behavioral indicators. To further investigate these associations, it is essential to examine the correlation between neural activity, such as hemodynamic responses (e.g., fNIRS or fMRI) and electrophysiological signals (e.g., EEG), to determine whether similar patterns are observed at the neural level.

### Conclusion

Our study suggests that cognitive functions may play a more critical role than non-cognitive factors in explicit decision-making. Among the components of cognitive function, attentional inhibitory control was particularly associated with appropriate decision-making. This result indicates the importance of focusing on cognitive abilities when aiming to promote advantageous decision-making under risk. Future research will require larger sample sizes and the incorporation of additional factors that may influence decision-making, such as neural activity and physiological responses.

## Supplementary Materials

The behavioral data and analysis syntax used in this study are available on the Open Science Framework (OSF; see Yoshimaru, 2025).



YoshimaruY.
 (2025). Behavioral_data
[Data, syntax]. PsychOpen. https://osf.io/xg8q3


## Data Availability

For this article, the data and syntax are freely available (see Yoshimaru, 2025).
